# Unmasking the complexities of healthcare access in low-resource settings: a health systems approach to obstetric and under-5 healthcare in rural settings of Eastern Uganda

**DOI:** 10.1080/16549716.2024.2397163

**Published:** 2024-09-09

**Authors:** Rornald Muhumuza Kananura

**Affiliations:** a African Population and Health Research Center, Nairobi, Kenya; b Centre of Excellence for Maternal, Newborn and Child Health, Makerere University School of Public Health, Kampala, Uganda; c School of Economics and Political Science, Department of International Development, Houghton St, London, UK

**Keywords:** Obstetric, child, healthcare access, Uganda, sub-Saharan Africa

## Abstract

**Background:**

Access to appropriate obstetric and under-5 healthcare services in low-resource settings is a challenge in countries with high mortality rates. However, the interplay of multiple factors within an ecological system affects the effectiveness of the health system in reaching those in need.

**Objective:**

This study examined how multiple factors concurrently affect access to obstetric and child healthcare services in resource-poor settings

**Methods:**

The research used social autopsies [in-depth interview] with mothers who experienced newborn death [*n* = 29], focus group discussions [*n* = 8] with mothers [*n* = 32], and fathers [*n* = 28] of children aged 6–59 months, and the author’s field observations in Eastern Uganda’s rural settings. The research employed narrative and inductive thematic analysis, guided by concepts of social interactions, behaviour, and health institutional systems drawn from system theory.

**Results:**

The study unmasked multiple concurrent barriers to healthcare access at distinct levels. Within families, the influence of mothers-in-law and gender dynamics constrains women’s healthcare-seeking autonomy and agency. At the community level, poor transport system, characterised by long distances and challenging road conditions, consistently impede healthcare access. At the facility level, attitudes, responsiveness, and service delivery of health workers critically affect healthcare access. Negative experiences at health facilities profoundly discourage the community from seeking future health services.

**Conclusion:**

The findings emphasise the persistent influence of structural and social factors that, although well documented, are often overlooked and continue to limit women’s agency and autonomy in healthcare access. Enhancing universal access to appropriate healthcare services requires comprehensive health systems interventions that concurrently address the healthcare access barriers.

## Paper Context

- **Main findings:** The findings highlight the persistent influence of structural and social factors that have been well documented yet often overlooked.
- **Added knowledge:** While the barriers to and essential components of an effective health system are well known, the current global health space requires an understanding of how the structural, cultural, and social nuances have persistently affected the marginalised communities.
- **Global health impact for policy and action:** For a transformative health system, promoting equitable and accessible appropriate healthcare for all necessitates a holistic approach that identifies and addresses healthcare access barriers.

## Background

The Global Sustainable Development Goal 3 [SDG-3] aims to reduce newborn and under-five mortality to less than 12 and 25 per 1000 live births, respectively [1]. However, given the current progress in reducing newborn and under-five mortality in sub-Saharan Africa [SSA] countries, achieving these SDG-3 goals appears ambitious for the region. Despite the significant reduction in under-five mortality from 148 deaths per 1000 live births in 2000 to 46 in 2019, the rates remain unacceptably high [2]. Notably, newborn mortality [death within the first 28 days of life] accounts for the largest share [53%] of under-five mortality in SSA [3]. Even in Uganda, like other SSA countries, new-born mortality has remained stagnant at 27 deaths per 1000 live births over the last two decades [4,5].

Timely access to appropriate obstetric services could reduce newborn mortality by 41% and stillbirths by 70% in high-burden countries [6]. Nevertheless, access to care for women during labour and for sick children in Uganda continues to be a challenge. Approximately 30% of deliveries in Uganda occur outside formal health institutions and without skilled professional assistance [5]. Similarly, less than half [48% and 39%, respectively] of children with fever and suspected pneumonia seek care within 24 h [5]. To achieve the 2030 SDG-3 national commitment of reducing newborn and under-five mortality [7], it is essential to focus on increasing access to appropriate care.

However, healthcare access is a complex interplay of social and structural factors within an ecological framework [individual, family, and community] [8,9], which affects the effectiveness of the health system in reaching those in need. Factors such as family networks, socioeconomic status, and individual-level factors such as marital status, education, age, and wealth influence access to obstetric and child health services at the family level. At the community level, aspects, such as community structure, functioning, societal norms, quality of leadership, and availability of social services, including hospitals and civil society, shape human behaviour and decisions to access health services [9]. Moreover, health facilities in resource-poor settings are often inadequately equipped to provide the needed obstetric and child health services [10], leading to lower patient confidence and trust in healthcare access. The presence of these barriers and factors can affect families and individuals differently, hindering them from accessing appropriate healthcare services. In resource-poor settings, families and individuals often encounter multiple concurrent barriers, such as poor transport, financial constraints, and inadequate health supplies, which affect their access to appropriate services. However, available studies overlook how structural barriers and social factors, including the diverse range of social agents, concurrently inhibit the population from accessing appropriate healthcare services.

Guided by the concepts of social interactions, behaviour, and health institutional systems drawn from systems theory [11–14], the study aims to bridge this gap by examining how multiple factors concurrently affect access to obstetric and child healthcare services in resource-poor settings. Additionally, it seeks to challenge the tendency to blame individuals or families by considering the broader community context, including power dynamics, that shape healthcare access. Furthermore, the findings of this study contribute to the importance of health systems interventions that address the multifaceted challenges of obstetric and child healthcare access in resource-poor settings. They underscore the need to recognize the considerable influence of context in the design of interventions that improve access to appropriate healthcare.

## Methods

### Study design and setting

The data for this study were collected through qualitative multimethod research conducted between 20 August 2019, and 12 December 2019, in four villages located in the Iganga and Mayuge districts of central-eastern Uganda. These predominantly rural districts have a health staffing level of 71%, aligning with the national average of 73%. The Iganga and Mayuge districts have 49 and 55 health facilities, respectively, serving an average population of 500,000 each. Approximately 18% and 15% of health facilities in Mayuge are privately owned clinics, while in Iganga, they are approximately 20% and 24%, respectively. Approximately 27% and 15% of health facilities in Iganga and Mayuge, respectively, offer obstetric services, either government or private not-for-profit facilities.

Uganda’s health service providers include traditional therapies and formal healthcare providers at various levels [15]. Approximately 30% of the recognized formal health facilities are beyond the recommended 5 km distance, affecting access for certain communities [16]. Additionally, approximately 30% of health worker positions are vacant, and absenteeism among health workers is estimated at 44.7% [17].

## Data collection

Participants were mobilised at the community level with the help of village health team [VHT] members in Uganda. In-depth interviews [IDIs] were conducted with 29 women who recently delivered and experienced stillbirth or newborn death. IDI participants were identified through VHT records and approached by VHTs to explain the study and gain their voluntary participation. All selected women agreed to participate, and interviews took place in their homes, ensuring privacy by excluding family members. Permission was sought from married women’s partners, and no participant declined. Two female research assistants conducted interviews in the Lusoga language, with the author observing three interviews. The interviews lasted 30–40 min and were audio-recorded.

Focus group discussions [FGDs] were conducted separately with mothers [4 groups] and their partners [4 groups] who had children aged 6–59 months. FGDs were held in secure locations, such as VHTs’ homes or community chairpersons’ homes, ensuring privacy. Participants collectively determined a suitable location. Each FGD had 6–8 participants, addressing topics related to treating sick children, pathways to accessing services, and perceptions of health service accessibility. A female research assistant conducted all the FGDs in the Lusoga language, and interviews lasted 50–60 min and were audio-recorded.

Participants provided informed consent, either by signing a consent form or by providing a thumbprint if they could not read. They were informed of their right to withdraw without giving a reason or answering uncomfortable questions. Copies of the consent form were given to participants, ensuring anonymity with study identification numbers and pseudonyms used instead of real names.

For all research participants, including the health workers, a nonmonetary gift [bar of soap] equivalent to 1 GPB [4500 Ugandan shillings for 1 GBP] was given. However, the gift did not coerce their participation since nothing was expected during enrolment and throughout the interview process.

## Data management and analysis

Transcripts were uploaded to RQDA, a qualitative data analysis package, for coding and framework analysis [18]. Open coding was performed as the author read through each transcript. Codes were indexed and organised into thematic categories, guiding the presentation and interpretation of results. Interview guides were designed to minimise bias and encourage participant reflection. The analysis followed two qualitative analysis approaches. Initially, the study conducted a narrative analysis approach to explore and comprehend the community lived experiences. Subsequently, the study applied an inductive thematic analysis approach, allowing participants’ voices and themes to emerge. Twenty-three codes were identified and grouped into three themes and eight subthemes (Table 1).

**Table 1.** Codes that were generated through open coding using RQDA.

Themes	Subthemes	Codes
I. Family and community social networks influence primary caregivers’ (women) autonomy	1. Existing family support networks	1. Husband 2. Grandparents 3. Family friends or neighbours 4. Community transporters
	2. Gender dynamics	5. Therapeutic provision
	3. Ways of support	6. Enforce Cultural values and practices. 7. Caretaking 8. Financial and material support
		9. Information and experience sharing about health services, treatment options
II. Role of Community System Characteristics	4. Social infrastructures or services	10. Community roads 11. Health facilities (quality and proximity)
	5. Leadership or political will(authority)	12. Transport services(ambulances) 13. Enforce behavioural change
	6. Community diagnosis and therapeutic system	14. Traditional birth attendants 15. Drug shops 16. Community health workers
III. Health services system factors	7. Health responsiveness 8. Community trust in health services	17. Respectful care 18. Service charges 19. Stock out of drugs 20. Health workers availability 21. Experience, and skills 22. Customer care 23. Patients’ experience with health institutions or health workers

## Reflexivity and reflection in data collection

This section presents a reflection of data collection procedures’ and participants’ involvement, considering ethical considerations for future studies. The study prioritised the emotional experiences of bereaved women during interviews and as such the two research assistants who conducted the IDIs had counselling training and experience in verbal autopsy. During the interactions, the research assistant demonstrated empathy by giving participants time to compose themselves when overwhelmed. Despite shedding tears, most participants expressed their willingness to continue with the interview and conveyed how the interaction reduced their grief. One woman shared her thoughts, saying, ‘I am incredibly happy that someone has reached me to ask for the reasons why my baby died. No one in this community has ever sat with me to ask me why my child died. I now feel relieved, and I hope what I have shared with you will help to improve the health of the community’.

Furthermore, during focus group discussions [FGDs], disruptions occurred due to children’s presence. To mitigate this, we provided refreshments, which helped in maintaining a conducive atmosphere for dialogue. Furthermore, FGDs highlighted the limited engagement of younger women, due to older women’s dominance.

## Findings

### Description of study participants

Tables 2 and 3 summarise the participants’ characteristics of in-depth interviews and focus group discussions. Overall, 23% of the women had recent deliveries conducted in the communities. Among the FGD female participants, 41% and 22% had ever experienced child loss and stillbirth, respectively. Among women who had recently experienced child loss [in-depth interview], 48% of the newborns had died within the first day of delivery, and all deaths occurred within the first 6 days of life.

**Table 2.** Interviewed women in the focus group discussion and in-depth interviews.

	Women with children aged 6-59 months – FGD		Women experienced recent child loss- IDI		Total	
	Freq.	Percent	Freq.	Percent	Freq.	Percent
**Marital status**						
Married or staying with a partner	29	90.6	29	100.0	58	95.1
Unmarried	3	9.4			3	4.9
**Age group**						
20–30	21	65.6	19	65.5	40	65.6
30–40	9	28.1	6	20.7	15	24.6
<20	2	6.3	4	13.8	6	9.8
**Education level**						
None	13	40.6	1	3.5	14	23.0
Primary level	11	34.4	16	55.2	27	44.3
Post-primary level	8	25.0	3	10.3	11	18
**Occupation**						
Civil servant	2	6.3	1	3.5	3	4.9
Business	4	12.5	7	24.1	11	18.0
Farmer or casual labourer	26	81.3	21	72.4	47	77.0
**Religion**						
Muslim	10	31.3	14	48.3	24	39.3
Protestant and Catholic	17	53.1	14	48.3	31	50.8
Others	5	15.6	1	3.5	6	9.8
**Ever experienced child or pregnancy loss**						
No	19	59.4	–	–	–	–
Yes	13	40.6	–	–	–	–
**Ever experienced stillbirth**						
No	25	78.1	–	–	–	–
Yes	7	21.9	–	–	–	–
**Gravida**						
0– 4	18	56.3	19	65.5	37	60.7
5+	14	43.8	10	34.5	24	39.3
**Place of delivery of the recent birth**						
Clinic	6	18.8	7	24.1	13	21.3
Formal Health facility	16	50.0	18	62.1	34	55.7
Community	10	31.3	4	13.8	14	23.0
**Age at death in days**						
0–1	–	–	14	48.2	–	–
2–6	–	–	15	51.8	–	–
**Birth weight of the child that died**						
2.5+ kg	–	–	19	62.1	–	–
<2.5 kg	–	–	10	34.5	–	–
Missing -	–	–	1	3.5	–	–

**Table 3.** Description of men who participated in the focus group discussion.

	Freq	%
**Education level**		
None	15	53.6
Primary level	10	35.7
At least a secondary level	03	10.7
**Primary occupation**		
Community transporters(Boda-boda)	15	53.6
Business	4	14.3
Farmer	9	32.1
**Religion**		
Muslim	13	46.4
Christian	15	53.6
**age group**		
20-30	15	53.6
30-40	7	25.0
41+	6	21.4
**Have multiple wives**		
No	21	75.0
Yes	7	25.0

## Thematic findings

**Figure 1.**
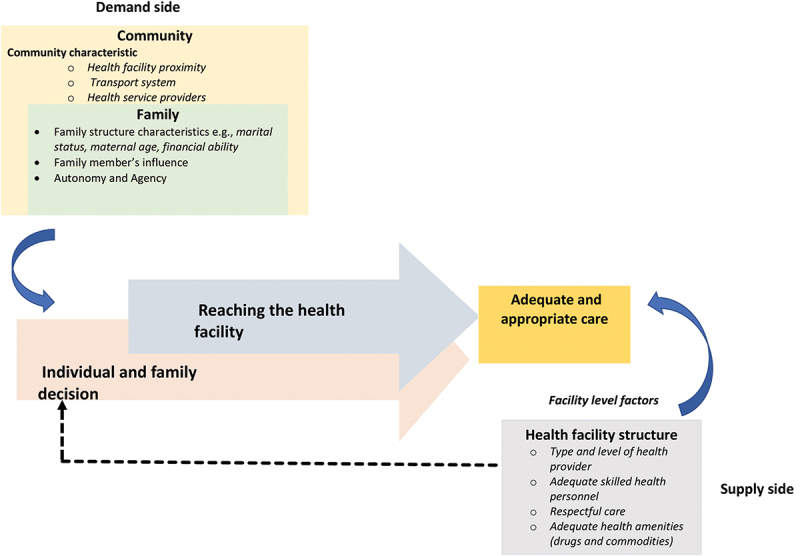
Interplay of multiple factors in shaping appropriate obstetric and under-5 healthcare access in low-resource settings.

The research identified three main themes that relate to family and community social networks’ influence on primary caregivers’ autonomy [theme 1], how community characteristics affect access to healthcare [theme 2], and how health facility system factors affect access to healthcare [theme 3]. Interview and field observation narratives are used to demonstrate how multiple concurrent factors affect appropriate access to obstetric and child healthcare services. Figure 1 provides a summary of how the themes overlap and interact to affect appropriate access to obstetric and child healthcare services.

### Theme 1. Family and community social networks influence primary caregivers’ [women] autonomy

Family networks provide financial support, act as caretakers, offer traditional therapies and have authority in the community. Husbands and mothers-in-law hold significant power over women’s autonomy, deciding when and where they seek healthcare and provide therapeutic care. An IDI participant shared an experience where her mother-in-law chose a traditional birth attendant instead of a higher-level facility for delivery, fearing a caesarean section.

‘We went to the health facility in this village [Lower health facility], and they [health workers] told us to go to Nakavule [referred to the higher-level hospital]. However, when we were on our way, my mother-in-law changed and decided that we go to the TBA since there might be a possibility of operating [undergoing a C-section] me at Nakavule. We went to the TBA and that’s where I delivered from.’ – **IDI mother participant**

In another IDI interview, a woman mentioned how her mother-in-law would bring traditional drugs that she was not familiar with, highlighting the impact of traditional practices on healthcare decisions. *‘…. She [meaning her mother-in-law] used to bring local herbs that I did not know’*. Additionally, mothers-in-law were found to influence behavioural practices such as exclusive breastfeeding and weaning, and they could also influence their sons [women’s husbands] on what they should provide for their wives, including access to health services.

In the study area, the interview narratives shed light on how caregiving for children and women is typically perceived as the husband’s responsibility. In the cultural context of the participants’ tribe [Busoga], mothers are seen as the primary caregivers, while fathers are often away from home for extended periods due to work commitments. Consequently, men are considered the main providers of financial resources within the family. One of the women FGD respondent narrated, *‘As a Musoga [relating to the culture of the participant’s tribe], let me say in Busoga [participant emphasizing their tribe’s culture] because it is where I have lived, I know that it is mainly men who have money’*. Participants emphasised that in Busoga culture, men are seen as the ultimate authority, and women usually depend on them for financial support. Thus, the financial capability of the husband or family determines where when, and what type of healthcare services can be accessed. As one of the women FGD respondent expressed, ‘*In Busoga, it is known that the father is the supreme and women often ask their husbands for money, and if the husband refuses to provide it, accessing necessary healthcare for the children and themselves becomes challenging’.*

Women must seek permission from their husbands on where and when to access healthcare services, thereby restricting their decision-making autonomy and agency. For example, if a child falls sick or a woman experiences labour contractions while the husband is away, they must wait until the husband returns home to decide on the appropriate course of action regarding healthcare. One of the women FGD participant shared her concern stating that, *‘It is the father you have to inform that I am taking the child to a health facility, but they [health facilities/workers] need some money. They [Fathers] do not have time to see if the child has bathed, how they have stayed, they are there to support us financially’.*

Traditional gender roles also extend to the responsibility of fathers in seeking healthcare for their children. Fathers are expected to take charge and provide financial support for their child’s healthcare needs. If the child falls sick, it is often the father’s role to visit the clinic and obtain medication or take the child to a health facility, if necessary, as one the women FGD participant recounted, *“… Musawo [referring to the interviewer as a health worker], he [husband] can stop you from going to the health facilities in case he doesn’t have money since most of us depend on them for financial support. Because he can say that ‘if you have the money, take the child but me I don’t have and so if you don’t have, that means that the child will not receive treatment at all.’*

Such stereotype gender roles were also in unison affirmed by men who participated in the FGD as one of them explained. *‘When you come back home, your wife will tell you that my friend, while you were away, this child fell sick, so when you ask what happened? She explains to you what transpired at that time when you were not around. So as a father, you have the role to go to the clinic and bring tabs for the child to become better. If it requires taking him to the health facility, then they go’.*

Furthermore, the sharing of previous experiences at health facilities by family networks can either encourage or discourage other network members from accessing health services in the same facilities. For instance, if a family member had a negative experience at a particular health facility, they may discourage others from seeking care there. They may recount stories of health workers being unbothered, providing inappropriate drugs or exhibiting rude behaviour. These accounts can demoralise individuals and lead them to avoid that particular health facility, fearing a similar unpleasant experience as one of the women FGD participant described. *‘ … you may tell someone that I want to take the child to this health facility, but they then tell you that my friend has ever taken there a child, but health workers there were unbothered or maybe she was given drugs from there that didn’t match with the disease and I gave her and the child failed to get healed, so since your child is suffering from a similar disease, do you think it will get healed? And then you also get demoralized by going there.’*

## Theme 2. The role of community system characteristics in accessing appropriate obstetric and child healthcare services

### Social and public infrastructures or services

The narratives from the interviews shed light on the critical role of well-equipped health facilities and a reliable transport system in ensuring access to healthcare services for women and children in resource-limited settings. Communities with good road networks and access to reliable transport services are more likely to have easier access to government hospitals. Women living close to such communities or district centres tend to prefer going to the hospital rather than lower-level health facilities. In contrast, those living in hard-to-reach areas, characterised by poor transport systems and limited access to transport services, face significant challenges in reaching health facilities. One of the women FGD participant narrated, *‘We have challenges because a child falls sick but when you do not have a motorcycle at home. You must look for one with it and you reach there, and they tell you, this night can I go to Iganga?’*. Iganga town center is approximately 8 km away from the village where the interview was conducted, further highlighting the challenges of healthcare access, particularly in rural areas with limited transportation options. The use of boda-bodas as the major means of transport in these areas was found to be uncomfortable, particularly for pregnant women in labour and sick children. Additionally, transport fares in these areas were reported to be exorbitant due to long distances and poor road conditions, making it financially burdensome for families. Most women shared the same concern as one of them narrated. *‘The situation I was talking about concerns the distance we have to the government health facility is a very long journey because it is in Busowoobi, and the other is at Bukwaya. To and from you may be charged between 5000 – 6000/= for a person, which is expensive for us.’*

The poor transport systems challenges were observed during fieldwork, where women and children got involved in motorcycle accidents while travelling to health facilities [**Appendix 1, Narrative 1**]. Another case involved a woman who travelled a long distance to deliver at a health facility and needed referral due to complications but was not promptly transported [**Appendix 1, Narrative 2**].

Interview narratives highlight the concurrent difficulties caused of poor transport systems and drug stockouts. Whenever essential drugs are unavailable, patients are referred to distant higher-level facilities, making referral processes difficult, especially with motorcycle transportation. A male FGD participant narrated. *‘The reason why children die, is, sometimes you get a child from home to the health facility, you reach the nearest health facility where you are meant to go and there are no necessary requirements for testing and treatment. Therefore, when there are no drugs that are going to treat the disease, he/she is suffering from, they refer you to a far place. In referring you to a far place/long distance, it is like you are using a motorcycle and the child dies along the way’.*

### Leadership or political will

Local politicians play a significant role in addressing healthcare challenges by mobilising resources and enforcing behavioural changes. For example, a member of parliament sponsored the provision of ambulance vehicles at health facilities for referrals, improving access to higher-level care. However, practical challenges exist, such as the need for fuel contributions, which may hinder poor individuals from benefiting as one male FGD participant narrated. *‘… we are a beneficiary of a committed member of parliament for this county. He provided us with two ambulances. However, we must contribute fuel and therefore those with no money to contribute towards fuel may not be transported’*. Community leaders also promote behavioural changes. For instance, making sure that every household has a toilet, addressing issues of domestic violence, and taking part in other health campaigns like vaccination.

### Community diagnostic and therapeutic providers

Community health service providers include community health workers, traditional birth attendants, traditional healers, and drug shop owners. Community health workers mobilise and educate families on child health and provide drugs and nutrition guidance. Traditional birth attendants aid pregnant women and newborns. In this study, 3 in 10 women participants delivered under the help of a TBA. The TBAs’ good customer service, financial constraints and long distance were emphasised as the reasons to why women delivering in their places. During the FGD, a female participant mentioned turning to a Traditional Birth Attendant [TBA] for assistance during financial hardships. *‘At the TBA, we go there according to the situation has hardened when the pocket is empty.’* Another IDI woman participants who experienced the death of a newborn, shared how she used a bicycle to travel to the Traditional Birth Attendant [TBA] due to a shortage of funds for a motorcycle to take her to the health facility. During the interview, she recounted, ‘*Labour started at approximately 2:00 am and my partner said now we don’t have money for a motorcycle, let me put the bicycle out and I take you to deliver from there at Namukuve’s [TBAs home]’.*

The interview narratives shed light on the prevalent belief in traditional therapies for certain diseases, leading to the exclusion of modern medical interventions when children fall ill. Participants shared that this knowledge is often passed down through generations from their ancestors or grandparents. Consequently, in some cases, sick children are removed from health facilities and brought to traditional and religious healers for treatment. During the female FGD, one participant confidently asserted, with the agreement of others, that when a child is suffering from a condition known as Biwala, they do not seek treatment at the hospital but rather turn to traditional healers. *‘There is when the child is suffering from Biwala and you know that it is Biwala, there you don’t go to the hospital, and you go to these others [traditional healers].’* Another participant added that in such cases, injection treatments at health centres are feared, as they believe it may lead to the child’s death. *‘Yes! That person must not be injected’*, and another one interjected, *‘At the health centre if the child is injected, it dies.’* Biwala was described by participants as a disease condition characterised by symptoms of acute diarrhea, fever, convulsions, and sores.

Furthermore, the study participants emphasised how the community rely on drug shops for treatment of their children. Participants explained how drug shop owners could provide treatment based on available funds, offering half doses to individuals with limited funds, and sometimes on credit. *‘You find a person who may have their 500/= and they ask for some Panadol, some coartem, and they mix for them like that. Even when they do not get the full dose, they get a quick relief as they wait for the morning.’*

## Theme 3. Health services system factors

Participants recounted factors affecting trust and responsiveness, including respectful care, service availability, customer care, patient experience, and health workers’ perceived skills. Negative experiences with health workers led some women to choose TBAs or local drug shops. The interaction with the community provided insight into the inefficient triage procedures within the health facilities. Several participants recounted instances where they brought children and women in serious conditions, only to have them left waiting without receiving attention. One participant shares a heartbreaking incident where a child died while queuing.

*‘I have ever experienced a child who died while at the health facility because of health workers’ delays and negligence. I got this child from this village of ours who was aged 3 years. We reached the health facility, and they just told us to queue up and later took us to a small room, but before entering, another patient was being worked on. We were then sent to another room where a health worker was working on a patient. We were told to wait, and this took a long time. We were in the facility, and the baby died in our hands without seeing anybody to work on us.’ –* **FGD male participant [Community transporter]**

Another IDI woman participant who had experienced newborn death narrated her worst experiences while at the health facility. *‘I was handled badly when I went for delivery. A health provider told me that she wanted 20,000/= before she could work on me, yet I never had the money by then’.*

Furthermore, adolescent mothers face neglect from healthcare workers during childbirth. One of the interviewed adolescent mothers [15 years old] who had experienced newborn loss shared her distressing experience, where despite arriving at a health facility, she abandoned. Desperately, the adolescent mother turned to a TBA, where she experienced labour complications that resulted to a newborn death.

“Immediately [after feeling labour pains], we with her mother] went to Madina [TBA within the village], but unfortunately, she was not around. I and my mother then got a boda-boda and went to the health facility. Reaching the health facility, the midwife looked at me and told me that since I was a teenager, she would not help me deliver in their health facility. She never even gave me a referral to another facility. Fortunately, the boda-boda rider knew a place [TBA] where his wife normally delivers – that is where he took us. When we reached, the woman [TBA] examined me and found that I had no blood. She then boiled the medicine that could give me the blood for me to be able to push. I took the medicines, and later, the labour pains increased, and I started pushing. I pushed, but I was too tired. The child was born alive but was also too tired. The child was too heavy and was not breathing well. After like 10 minutes, it stopped breathing.”

Drug stockouts at health facilities force women and caretakers to seek alternative treatment for pregnancies and sick children. Community members turn to local drug shops or traditional remedies instead of visiting facilities that they perceive as unhelpful. Health workers often instruct patients to buy needed drugs and supplies externally. Frustration is expressed by one of the FGD women participant.

… . you reach them [health workers] there and they register you, ask you for a book, once they ask for a book and examine the child to see what they are suffering from, they tell you what to buy, you have to buy drugs, syringe, the canular and all the requirements just like the person giving treatment sometimes … most of the time they need some money because Uganda is a corrupt country so there is nothing free. If you don’t have money, you can even lose your child. If you don’t have money, thinking that I will go to a government health facility and get free drugs, they are not there.

Such negative experiences at health facilities affect women’s willingness to seek future healthcare. An IDI participant who lost her newborn shared how her most distressing encounter during antenatal care influenced her choice not to return for delivery. *‘What prevented me from going to Facility A is that I went there when my stomach was paining, thinking I was feeling labour pains, we stayed in the queue, and the health worker who found me there said you mean you are feeling more pain than the others you have found there? That is why when I went to labour and went to the TBA’s place, I never bothered myself with going there’.*

Similarly, another IDI participant who had experienced newborn death expressed her reluctance to return to Facility X after experiencing an unfavourable interaction with the healthcare workers there. She attributed her decision to the haughty demeanour of the health workers, whom she felt looked down upon patients and treated them as though they were worthless. Such negative encounters left her feeling disheartened and discouraged her from seeking healthcare at that facility again. *‘What made me not go back to Facility X was because the workers were proud. The way they look at a patient as although we were useless’.*

## The interplay of healthcare access barriers

The narratives gathered during this study illustrate the complexity and interconnected nature of barriers to healthcare access. One of the IDI participants who had experienced newborn death shared a disconcerting account that illustrates this complexity. While in labour and having reached the health facility, she did not receive prompt assistance due to an unresponsive health facility system. *‘She [midwife] came and found me when I was trying to push and encouraged me to push. However, the baby’s head came out, but the shoulders could not come out. I pushed as the midwife insisted I should until I lost energy. The midwife did not help, and later, the baby died while still in my legs. It never came out. Later, she told us to go to the higher-level hospital in Iganga, but there was no means of transport. My husband had to call his brother who drove a truck that he brought and was put behind in the trial.’*

This narrative underscores the critical role of health facility responsiveness, interconnection with challenges of transportation and poverty, collectively shape birth outcomes.

In another instance, a participant in women FGD recounted how financial constraints and perceived negligence by healthcare providers acted as deterrents, deterring women from seeking care at medical facilities. The apprehension of being overlooked and the concern over unaffordable medical services led some to opt to stay at home, even when faced with potential risks. *‘For some of us, we do not deliver in health facilities because of our poor financial status. We fear that health workers will ask for money to pay for their services when we are not ready. We also fear being neglected by health workers. When we go to the health facilities, those health workers look at us as if they don’t know what we as mothers are going through. Therefore, we decide to remain at home where we know we would get some care from the relatives rather than go to the hospital to be neglected.’*

This narrative underscores the complexity interplay between financial barriers, community feelings of healthcare provider attitudes, and the decision-making process regarding healthcare-seeking behaviours.

## Discussion

This study adopts an integrated approach, combining health system, social, and family elements, to comprehensively investigate obstetric and child healthcare access in resource-limited settings. The study findings show that healthcare access in resource-limited settings is influenced by multiple intersecting factors occurring at distinct levels: family, community, and health facility.

At the family level, family networks, particularly mothers-in-law, have the power to shape healthcare-seeking behaviours and decisions of women [19–21]. These networks not only provide financial support and act as caretakers but also offer traditional therapies and possess authority within the community, enabling them to influence women’s choices regarding healthcare [19–21]. Furthermore, family networks shape healthcare-seeking behaviours through the sharing of previous experiences at health facilities. Negative experiences recounted by family members can discourage others from accessing health services in the same facilities, while positive experiences can serve as endorsements. Furthermore, consistent with other studies in the same settings [22], the research identifies traditional gender dynamics within families. These dynamics highlighted the predominant role of men as the primary providers of financial resources, consequently influencing women’s agency in seeking healthcare. The influence of family support and power over resources often compels women to follow the suggestions of their social support network, even if the suggestions contradict recommended health practices. The lack of autonomy may lead to the tolerance of family network abuses to maintain family solidarity and support. To promote equitable access to healthcare services, it is crucial to challenge cultural norms and empower both genders are essential in fostering women autonomy and agency in healthcare access [22–24].

At the community level, the study identifies recurring stockouts of drugs at the health facilities and poor transport systems and infrastructure such as long distances between health facilities and rural communities as key barriers to healthcare access, which is consistent with other studies conducted in similar settings [25]. Access to these infrastructures influences individuals’ healthcare-seeking behaviours, with good road networks and access to reliable transport services increasing the likelihood of accessing government hospitals. On the other hand, communities with poor transport systems face significant challenges in reaching health facilities. The use of boda-bodas as the main means of transportation in these areas poses discomfort and safety risks [26]. Poor transport systems also result in high transport fares due to long distances and poor road conditions, creating financial burdens for families. The observed accidents during fieldwork underscore the challenges of inadequate transportation infrastructure. The study emphasises how improvement in public investment in public infrastructure such as better roads and health facilities as well ensuring drug availability in health facilities is critical in reducing healthcare access barriers.

Furthermore, leadership and political will are identified as crucial factors in addressing healthcare access challenges in resource-limited settings. Some politicians have played an instrumental role in mobilising resources and implementing health interventions [27]. Health facilities should aim at disseminating health information to different authorities, including politicians, as a way of mobilising their support and making them accountable to the communities they serve [27].

Community health service providers, including community health workers, traditional birth attendants, traditional healers, and drug shop owners, play essential roles in providing healthcare services and guidance. Community health workers are highlighted as valuable resources who actively engage and educate families about child health and general well-being [28–30]. Traditional birth attendants and healers also have significant roles, attributed to positive reputations and negative perceptions of formal healthcare services in some communities [31,32]. Financial constraints sometimes lead women to seek assistance from traditional birth attendants instead of formal health facilities.

Furthermore, the study participants indicated how they rely on drug shops for treatment due to proximity, flexible payment options, and credit services. Drug shops adapt treatment options based on the availability of funds, providing at least some relief for health issues. However, while drug shops offer convenience and accessibility, they may not offer comprehensive care [33,34]. Collaboration between drug shop owners and formal healthcare providers can improve coordination, appropriate referrals, and ultimately better health outcomes [34–36].

At the health facility level, consistent with other studies [10,37,38], this study emphasises the critical importance of health workers’ attitudes, responsiveness, and efficient service delivery within health facilities. The narratives shared by participants shed light on the severe consequences of prolonged waiting times and dismissive behaviour exhibited by health workers towards patients and their families. The study results reveal a distressing situation where caretakers of children and pregnant women must endure long waiting periods while health workers deal with other patients. Tragically, in one instance, this delay and lack of prompt care resulted in the devastating loss of a child’s life, leaving the family deeply traumatised.

In addition to the issue of prolonged waiting times, the study also reveals that drug stockouts are a significant barrier to healthcare access, which results in communities’ reliance on drug shops and traditional remedies. Participants expressed frustration over the lack of accessible and affordable healthcare, which often compelled them to resort to traditional therapy and drug shops due to the shortage of medications in public health facilities. Referring patients to buy drugs and other amenities reflects a widespread belief among community members that public health services may not truly be free [33,39,40]. Addressing the issue of drug stockouts and ensuring the availability of essential medications in health facilities is crucial in addressing catastrophic expenditure arising from out-of-pocket.

Another concern highlighted by this study is the reluctance of health facilities to provide services based solely on the mother’s age, reflecting a deeply troubling issue of discrimination and lack of empathy towards vulnerable populations, such as adolescent mothers [41]. This study highlights the severe consequences of neglect and inadequate care faced by adolescent mothers in these resource-limited settings. This negligence illustrates the urgent need for a more inclusive and compassionate approach to maternal healthcare, particularly for adolescent mothers. Healthcare systems in such settings must prioritise sensitivity and respect when providing services, recognising the rights and needs of all individuals, irrespective of their age or background.

## Study strengths and limitations

The strength of the study is that it considers both social and health institutional factors, providing a comprehensive examination of obstetric and child healthcare access, offering a holistic understanding of the complexities involved in accessing healthcare services. Additionally, the use of IDIs with parents who had experienced child loss and FGDs parents of children aged 6–59 months allow for rich and nuanced insights into the lived experience of how multiple factors shape access to appropriate healthcare services.

Nonetheless, the study also has some limitations that are majorly aligned to the methodological implications. While women exhibited a keen willingness to share narratives detailing the circumstances surrounding the loss of their newborn, there was a need for psychosocial referral support, which was not provided. As pointed out in other studies, these results underscore the need for community-based psychological support for bereaved mothers, including those who have experienced stillbirth [42–44] to help the mothers cope with their loss and navigate the grieving process.

## Conclusion

The study findings emphasise the persistent influence of structural and social factors that have been well documented yet often overlooked. The findings underscore the consistent impact of structural and systemic violence on healthcare access. The entrenched gender dynamics and familial influences that limit women’s agency and autonomy in seeking healthcare perpetuates systemic violence where women must tolerate abuses to maintain family solidarity and support. The community-level barriers, such as poor transport system and substandard health services within the communities, perpetuates structural violence. The poor service delivery by health workers where individuals are subjected to inadequate care and health workers abuse reflects systemic violence. To promote equitable and accessible appropriate healthcare for all necessitates a holistic approach that addresses the identifies healthcare access barriers.

## Supplementary Material

Supplement.docx
