# Supplementary material for: Unmasking the complexities of healthcare access in low-resource settings: a health systems approach to obstetric and under-5 healthcare in rural settings of Eastern Uganda
Source: Glob Health Action. 2024 Sep 9;17(1):2397163. doi: 10.1080/16549716.2024.2397163 (PMC11391869; doi:10.1080/16549716.2024.2397163)
Supplement: Supplement.docx [file ZGHA_A_2397163_SM8599.docx]

### **Narrative 1: Study area setting field observation case**

| This field observation case took place in August 2019 during the collection of qualitative interviews. It occurred in one of the villages in Kityerera Subcounty, located in the Mayuge district. My research assistant and I conducted interviews with women in this village. To reach the village, we were transported by an old taxi van, commonly used for public transport, from Mayuge town council to Kityerera trading centre. We were advised to wake up early in the morning as there was only one taxi that typically departed early to Kityerera during the time of our study. In case we missed the taxi, our alternative means of transportation would have been a boda-boda (a motorcycle taxi). The road used for data collection was unpaved and rough at the time, stretching approximately 30 km from Mayuge town council to Kityerera trading centre. From Kityerera, the distance to our specific research location was around 5 km, and the means of transport available (during data collection) were either by boda-boda or bicycle. Due to the high demand for fuel, there were several makeshift "petrol stations" in the form of stalls or kiosks, storing the fuel in jerrycans and selling it in 300ml bottles for 3,000 Ugandan Shillings.  The area has poor road conditions, and during the rainy season, it can be challenging to travel on a boda-boda. For example, during our journey to the village, we witnessed an accident involving a woman and her five-year-old child who were traveling on a boda-boda. The accident was caused by the bumpy and slippery road due to the rain. All three individuals, including the rider, the woman, and the child, sustained injuries. Upon inquiring about their destination, the woman informed us that she was taking her child to the health facility for treatment of fever symptoms. The distance from the accident site to the health facility was approximately 4 km. The nearest public health center level IV is situated 30 km away from the interview venue.  Additionally, both Iganga and Jinja general hospitals are also located 30 km away from the same health center. This suggests that accessing specialized services provided by these health facilities may present difficulties. As we tried to return to our place of residence in the evening, we encountered difficulties in finding any available vehicle. The only transportation option was a boda-boda, despite the rain. This scenario highlights how limited access to transport options and poor road conditions can hinder access to health services, even when the recommended distance to a health facility is within 5 km. |
| --- |

### **Narrative 2. Health facility’s setting field observation case**

| This fieldwork observation case was documented in September 2019 while conducting interviews with the health workers at one of the facilities. While interviewing the health workers, later (after 30 minutes into the interview), we had someone calling [non-medical health worker] – *“Musawo*^[[1]](#footnote-1)^ *Jessica, there is a woman in labour that has arrived. She seems to be in critical condition”,* she said. Immediately “Musawo Jessica” left to attend to her, and we remained around observing^[[2]](#footnote-2)^. The patient had travelled with a female caretaker who was her family relative. I asked the caretaker to describe how the labor had started, and she explained that it began in the morning around 11:00 pm. However, they delayed coming to the health facility because it was nighttime and they did not have any means of transportation. *“The labour started in the morning at around 11:00 pm, but we delayed coming to the health facility because it was at night, and we did not have any means of transport”,* she said. Additionally, they did not have enough money for transportation as their village was quite a distance from the facility, so they had to walk slowly. *“Even then, we did not have enough money for transport because our village is quite a distance away from this facility, so we had to walk, and when you are in labour, you have to walk slowly”,* she added. They eventually arrived at the health facility around 11:00 am.  The midwife at the facility worked diligently to ensure a successful delivery for the mother. Unfortunately, the mother experienced postpartum haemorrhage, which required her to be referred to the district hospital. However, the facility did not have any means of transportation for referrals. The caretaker began contacting family members and making phone calls to gather money for transportation (I overheard her speaking to different people). She spent at least 30 minutes at the facility making these calls before transportation was arranged.  Eventually, they were transported to the district hospital using a boda-boda owned by a Village Health Team (VHT) member affiliated with the facility. With the assistance of nurses and VHTs, the patient was carefully lifted onto the boda-boda for transportation, with the caretaker sitting behind carrying the baby. The patient was in clear distress, unable to open her eyes or speak due to the intense pain she was experiencing.  The distance from the facility to the hospital was approximately 15 km. After conducting some additional research work, we followed up on this case with the health worker and the VHT member who transported the patient. We were informed that when they arrived at the hospital, there was a shortage of blood, necessitating her referral to the regional hospital. However, the family had to first raise money by selling some belongings, which caused further delays. Sadly, the woman passed away, leaving behind her newborn baby. This case highlights multiple barriers that hindered appropriate access to healthcare services. |
| --- |

1. Meaning health worker [↑](#footnote-ref-1)
2. We suspended the interview since the health worker became engaged with the patient. [↑](#footnote-ref-2)
